# Clustering of Largely Right-Censored Oropharyngeal Head and Neck Cancer Patients for Discriminative Groupings to Improve Outcome Prediction

**DOI:** 10.1038/s41598-020-60140-0

**Published:** 2020-03-02

**Authors:** Joel Tosado, Luka Zdilar, Hesham Elhalawani, Baher Elgohari, David M. Vock, G. Elisabeta Marai, Clifton Fuller, Abdallah S. R. Mohamed, Guadalupe Canahuate

**Affiliations:** 1 University of Iowa, Department of Electrical and Computer Engineering, Iowa City, 52242 USA; 2 University of Minnesota, Division of Biostatistics, Minneapolis, 55455 USA; 3 University of Illinois at Chicago, Department of Department of Computer Science, Chicago, 60607 USA; 4 MD Anderson Cancer Center, Department of Radiation Oncology, Houston, 77030 USA

**Keywords:** Data mining, Machine learning

## Abstract

Clustering is the task of identifying groups of similar subjects according to certain criteria. The AJCC staging system can be thought as a clustering mechanism that groups patients based on their disease stage. This grouping drives prognosis and influences treatment. The goal of this work is to evaluate the efficacy of machine learning algorithms to cluster the patients into discriminative groups to improve prognosis for overall survival (OS) and relapse free survival (RFS) outcomes. We apply clustering over a retrospectively collected data from 644 head and neck cancer patients including both clinical and radiomic features. In order to incorporate outcome information into the clustering process and deal with the large proportion of censored samples, the feature space was scaled using the regression coefficients fitted using a proxy dependent variable, martingale residuals, instead of follow-up time. Two clusters were identified and evaluated using cross validation. The Kaplan Meier (KM) curves between the two clusters differ significantly for OS and RFS (p-value < 0.0001). Moreover, there was a relative predictive improvement when using the cluster label in addition to the clinical features compared to using only clinical features where AUC increased by 5.7% and 13.0% for OS and RFS, respectively.

## Introduction

Every year over 50,000 new cases of head and neck cancers are diagnosed in the United States. This number is projected to rise in the future, especially for oropharyngeal cancers, recently been associated with the incidence of HPV16 genotype infections^1^. The American Joint Committee on Cancer (AJCC) and the Union for International Cancer Control, maintains an internationally used standardized TNM Staging System. This system serves as a way to systematically assess the severity of the cancer on individual subjects^2^. The vast majority of risk stratification of head neck cancer patients uses staging systems that sub classify patients into four or less groups, based primarily on committee derived treatment standards and approaches using existing data sets. These consider physical examinations, imaging and laboratory tests, pathology and surgical reports, etc. Establishing the AJCC stage for a patient considers various important anatomic classifications and other risk factors that contribute to the overall assessment such as T, N and M Categories. T Category relates to the extent of the primary tumor, N Category relates to the spread to lymph nodes, and M Category indicates the spread outside the T and N related areas. These classifications play a critical role in the ultimate diagnosis and prognosis. The ability to more accurately assess the underlying condition such that it improves the prediction on various outcomes is a long-standing clinical goal.

In the era of personalized cancer medicine, innovative sources of meaningful data are critically needed. For head and neck cancer, radiomics is one such “big data” approach that applies advanced image refining/data characterization algorithms to generate imaging features that may be used to quantitatively classify tumor phenotype in a noninvasive manner^3^. However, given the high number of radiomic features, extracting or identifying meaningful radiomic signatures is an active area of research^4–6^. Efforts to sub classify patients using novel imaging techniques will require infrastructure and conceptual approaches sufficient for incorporating these model parameters and thus are a significant unmet need for clinicians and informatics. Combining innovative data sources with a multitude of clinical features such as self-reported demographic information (e.g. race, sex, etc.), physician assessed categorizations (e.g. T Category, N Category, etc.) and other Electronic Health Record (EHR) data (e.g. patients medical history, lab and test results, etc.) is paramount for personalizing treatment. As part of a larger effort at implementing precision medicine approaches for oncologic care and head neck radiotherapy, radiomics features have demonstrated utility for discrimination of local regional recurrence^7^; in this effort we have extended this approach and shown stability across a series of risk stratification techniques, such as standardized AJCC values, in order to illustrate the difficulties and potential solutions spaces of incorporating radiomics for predicting global oncologic variables.

Machine learning is not new to cancer research. Artificial neural networks (ANNs) and decision trees (DTs) have been used in cancer detection and diagnosis for over 30 years^8–10^ and more recently Random Survival Forests^11^ (RSF) have been introduced. Initially, machine learning methods were used to identify, classify, detect, or distinguish tumors and other malignancies. In other words, machine learning was primarily used as an aid to cancer diagnosis and detection^12^. More recently, cancer researchers have applied machine learning towards cancer prediction and prognosis. Numerous machine learning (ML) methods have been adapted for survival analysis, prognosis, and prediction^13–15^. Machine learning algorithms are often classified on the basis of the desired outcome of the algorithm^16,17^. In supervised learning algorithms, a labeled set of training data or examples is used. In unsupervised learning, a set of examples are given, but no labels are provided. Clustering analysis is a type of unsupervised learning, where the goal is to find meaningful and or useful groups in the data^18^. A survey of clustering algorithms can be found in Xu and colleagues^19^ and of clustering in high-dimensional data in Kriegel *et al*.^20^. It is this analysis that we combine with the more traditional supervised methods to effectively capture meaningful groups with respect to the outcome. Several other groups of successfully implemented approaches, albeit without as elaborate investigation into feature stability and selection models^21,22^. Aerts *et al*. have reduced a large-scale lung data set with specific radiomics feature which could be cross applied to head neck cancer patients^23^. The same group subsequently led a comparative investigation into various machine learning approaches^24^. These approaches are of significance and informed our current approach, allowing us to provide extension of their binary classification with the utilization of survival data. Further, we compare against approaches which interrogate the additive value of scalable feature selection against both clinical variables as well as random forest based approaches. In this sense our work shows potential scalability to other non-head neck organ sites and serves as a workflow template for future prospective efforts designed for repeated classification and model improvement over time.

To illustrate the applicability of the proposed approach we consider two outcomes: overall survival (OS), and recurrence free survival (RFS) which is a combination of loco-regional (primary site recurrence of tumor or recurrence at lymph nodes) and distant control (distant metastases). These outcomes are said to be right-censored because for some patients the time-to-event may be unknown. This is the case for patients where the outcome has not been observed up to the last known follow-up time. Right-censored data poses challenges to training methods, especially those that require a known target. Nevertheless, the patients that have yet to incur an event can still provide us some useful information in order to predict the probability of an event occurring at a certain time. Survival analysis often attempts to use these right-censored outcomes in a meaningful way rather than discarding them.

In this work, the goal is to identify and exploit any underlying latent characteristics that may help stratify the feature space meaningfully towards some outcome. The proposed approach combines supervised and unsupervised methods such that ultimately clustering can be used to improve prediction of our outcomes of interest in the context of right-censored oropharyngeal head and neck cancer data. Since clustering is agnostic to the outcome, we first transform our feature space in order to relate the discovery towards the outcome. To achieve this the approach is straightforward, we first create a proxy dependent variable, the martingale residuals, then train a supervised model (such as linear regression) and ultimately use it’s fitted feature coefficients to scale the feature space towards the outcome. We evaluate the resulting groups through model comparisons of using its group label as a feature in a Cox Proportional Hazards (Cox) model considering Akaike Information Criterion (AIC) and the likelihood ratio test (LRT), and additionally by evaluating Kaplan Meier (KM) curves. Finally, we further evaluate the predictive performance against two common techniques in survival analysis, Random Survival Forest (RSF) and Cox by comparing on various metrics. These metrics are the area under the curve (AUC), Brier, concordance index C-Index) and calibration.

To summarize, the aims of this study are as follows: (1) incorporate outcome information to influence cluster analysis; (2) identify discriminative clusters using patient characteristics available at the time of diagnosis and radiomic signatures; (3) use the cluster labels to stratify the patients and generate KM curves for each cluster, and compare to AJCC stage; and (4) evaluate the predictive performance of including the cluster label as a feature in a Cox model and RSF for OS and RFS outcomes.

## Methods and Materials

All analyses were conducted using R version 3.4.1 (R Foundation for Statistical Computing, Vienna, Austria). All statistical tests are two-sided with statistical significance defined as a *p* < 0.05.

### Data

Patients were retrieved from an internal University of Texas MD Anderson Cancer Center database after getting approved by the University of Texas MD Anderson Cancer Center Institutional review board (IRB). All methods for this study were performed in accordance with the University of Texas MD Anderson Cancer Center IRB guidelines and regulations. This is a retrospective study approved by IRB, informed consent was waived as it is a retrospective study and in compliance with the Health Insurance Portability and Accountability Act (HIPAA) and IRB also approved the waiver of the informed consent.

The dataset consists of 644 of oropharyngeal cancer (OPC) patients who were treated at MD Anderson Cancer Center between 2005 and 2013. Following IRB approval, clinical features including age at diagnosis, sex, ethnicity, HPV status, smoking status and frequency, subsite of the primary tumor within the oropharynx, T category, N category, therapeutic combination and AJCC stage (7^*th*^ and 8^*th*^ edition) were extracted from electronic medical records. Table 1 shows the demographics of patients for the clinical features and survival outcomes considered. Summary measures of the distribution of the followup time and the proportion of censored is given in Table 2. A more detailed description of these data can be found in Elhalawani *et al*.^25^.

**Table 1:** Characteristics of study population.

Total Samples: 642	Median or Frequency	(25th, 75th centiles) or Percent	Missing Frequency (Percent)
Female			0
No	565	88	
Yes	77	12	
Age	58	(52.3, 65.3)	2 (0.3)
HPV Status			0
Negative	50	7.8	
Positive	391	60.9	
Unknown	201	31.3	
T Category			0
T1,T2,Tis,Tx	408	63.6	
T3,T4	234	36.4	
N Category			0
N0, N1	339	52.8	
N2, N3	303	47.2	
Smoking Status			0
Current	139	21.7	
Former	238	37.1	
Never	265	41.3	
Smoking Pack Per Year (Current)	35	(20, 50)	13 (2)
Tumor Subsite			0
BOT	328	51.1	
GPS, NOS, Soft Palate	57	8.9	
Tonsil	257	40	
White/Caucasian			0
No	56	8.7	
Yes	586	91.3	
Therapeutic			0
CC	339	52.8	
IC_and_CC	160	24.8	
IC_and_Radiation	61	9.5	
Radiation	82	12.8	
F25.ShapeVolume	7.7	(3.8, 14.8)	84 (13.1)
F29.IntensityDirectLocalRangeMax	1136	(1103, 1195.8)	84 (13.1)
F5.IntensityDirectGlobalMax	1199	(1165, 1341.8)	84 (13.1)
F29.IntensityDirectGlobalMax	1190.5	(1152, 1369.5)	84 (13.1)
AJCC 8th (Imputed with 7th ed)			2 (0.3)
I	238	37	
II	109	16.9	
III	74	11.5	
IV	221	34.3	

Following AJCC standard definitions, T1 - T4: “Size and/or extent of the primary tumor”, Tx: “Primary tumor cannot be evaluated”, Tis: “Early cancer that has not spread to neighboring tissue”, and N0-N4: “Involvement of regional lymph nodes”. BOT: Base of Tongue. NOS: Not otherwise specified. GPS: Glossopharyngeal Sulcus. CC: Concurrent Chemotherapy. IC: Induction Chemotherapy.

**Table 2:** Outcomes summary.

	Median or Frequency	(25th, 75th centiles) or Percent	Missing Frequency (Percent)
**Recurrence Free Survival**			6 (0.9)
Folow-up Time	61.1	(39.7, 96.2)	
*Censor Status*			
Censored	518	80.7	
Uncensored	118	18.4	
Event Time (Among uncensored observations)	17.5	(9.7, 37)	
**Overall Survival**			2 (0.3)
Follow-up Time	65.3	(45.6, 98.4)	
*Censor Status*			
Censored	510	79.4	
Uncensored	132	20.6	
Event Time (Among uncensored observations)	35.3	(16.5, 64.8)	

For imaging data, contrast-enhanced computed tomography (CECT) at initial diagnosis -prior to any active local or systemic treatment- were exported to a commercially available contouring software (Velocity AI v3.0.1). The volumes of interest (VOIs) including the gross primary tumor volumes (GTVp) were manually segmented by a radiation oncologist in 3D fashion, then inspected by a second radiation oncologist. The generated VOIs and CT images were exported in the format of DICOM and DICOM-RTSTRUCT to be used for radiomics features extraction. The primary tumor volumes (GTVp) were contoured based on the ICRU 62/83 definition^26^. Radiomics analysis was performed using the freely available open source software “Imaging Biomarker Explorer” (IBEX), which was developed by the University of Texas MD Anderson Cancer Center and utilizes the Matlab platform (Mathworks Inc, Natick, VA). The CT images in the format of DICOM and the GTVp contours in the format DICOMRTSTRUCT were imported into IBEX. We extracted features that represent the intensity, shape, and texture. The categorization of these features was ranked as first, second, and higher texture features based on the applied method from pixel to pixel^27^.

### Data preprocessing

Missing data were imputed using the Multivariate Imputation by Chained Equations (MICE) approach^28^. This is a standard approach widely used in data analysis. Predictive mean matching (with k = 5) was used for the imputation. Imputation of each validation sample was performed individually and only considering training after the training had been imputed, per fold. As we are comparing against AJCC stage, the 2 patients with missing values for it were discarded as were patients with missing response (2 for OS, 6 for RFS) times.

Min-Max normalization was used to standardize each attribute’s range into the interval [0, 1]. This was done as a pre- processing step for feature selection, model training, and clustering. This prevents features from dominating the dissimilarity value (e.g. Lp-norm) when clustering.

Out of the initial 3831 radiomic features, we removed those with zero variance and those with a correlation above 99%. Previous studies have identified tumor volume and intensity as relevant features for local control^7^. Moreover, physicians routinely use imaging for their assessment of the patient’s disease staging. As our goal is a data driven approach for patient stratification that improves survival outcome prognosis, we consider both clinical and radiomic features for clustering. To further reduce redundancy, we also removed any radiomic features that were highly correlated (>80%) to F25.ShapeVolume and F29.IntensityDirectGlobalMean. Finally, the RReliefF feature selector was applied over the remaining 542 radiomic features. The Relief family of algorithms calculate a feature importance value for each feature by calculating the distance between pairs of near observations which fall in the same and different classes^29^. Features with more similar values for observations having the same class get higher importance values and likewise features with more different values for observations not having the same class get higher importance values. RReliefF calculates feature importance based on a continuous outcome, in this case, the martingale residuals resulting from using a Cox model considering the clinical features. It achieves this by probabilistically determining whether the instances are different and is based on the relative difference between the outcomes.

Feature importance for the Relief algorithms in general is expressed by the following equation:

$$W[A]=P({\rm{d}}{\rm{i}}{\rm{f}}{\rm{f}}.\,{\rm{value}}\,{\rm{of}}\,{\rm{A}}|{\rm{near}}\,{\rm{instance}}\,{\rm{with}}\,{\rm{diff}}.\,{\rm{prediction}})-P({\rm{diff}}.\,{\rm{value}}\,{\rm{of}}\,{\rm{A}}|{\rm{near}}\,{\rm{instance}}\,{\rm{with}}\,{\rm{same}}\,{\rm{prediction}})$$

The number of iterations for the RReliefF algorithm was set to 1000. A radiomic signature of four features, described later in Results, was identified by the feature selection algorithm and included together with the clinical features (given in Table 1) for clustering. Given our evaluation of using the Cox model to assess the ultimate clustering, and comparing against this model using the original features, a reduced space of the entire radiomic feature space is necessary as otherwise there would be too many parameters for the Cox model to reasonably estimate.

### Multidimensional clustering

The clustering method applied in this paper is k-medians^30^. K-medians, as well as k-means, belongs to a family of k-centroid clustering algorithms^31^. In practice, these methods have proven very effective^32^. These partitioning clustering techniques are very popular, conceptually well understood, and with a solid statistical basis^30,33,34^. We decided to use k-medians given that many of the features are categorical, and the use of the median over the mean (as in k-means) is more robust to outliers^30^.

An iterative approach to performing k-medians is to initially set k samples as the initial cluster centers and identify them with an arbitrary label (i.e. initial “centroids”). Then the samples are associated to its nearest cluster as established by the dissimilarity, i.e. Manhattan distance in our implementation. After each iteration the centroids for each cluster are re-computed given the medians. Eventually the iterations converge, and these are ultimately the cluster labels assigned for k-medians.

In order to reduce the effect of the starting seeds selection and avoid local minima, we use consensus clustering^35^ to run k-medians 1000 times with different seeds and kmeans ++ initialization, in order to find consensus among the different iterations.

The consensus matrix is defined as:

$$ {\mathcal M} (i,j)=\frac{{\sum }_{h}{M}^{(h)}(i,j)}{{\sum }_{h}{I}^{(h)}(i,j)}$$

Where h is the h^*th*^ iteration of the chosen clustering algorithm. *I* and *M* are **N** × **N** matrices. *M* is the connectivity matrix where a cell is 1 if pair (i,j) appear together, 0 otherwise. And *I* is the indicator matrix where a cell is 1 if pair (i,j) are sampled for an iteration, 0 otherwise. Hierarchical clustering^32^ is then used on the consensus matrix to extract the clusters.

Validation sample assignment of cluster labels is done by computing the Manhattan distance to the centroids of the formed clusters and assigning the label of the closest centroid.

Cluster assignment per fold is arbitrary but may relate to the same underlying characteristic. Therefore, in order to visualize clusters and assess the cluster label assignment across folds, clusters at every fold are matched to fold 1 (arbitrarily selected). That is, if the training labels at a fold correspond with the training labels at fold 1 more than they don’t then the labels are kept the same, otherwise they are inverted. The validation samples are then assigned to these clusters. Given that the labels are arbitrary, this would just provide consistency of label assignment.

### Novel supervised scaling for clustering

Clustering without any considerations of the outcome data can certainly capture latent characteristics, but nevertheless these may not be related to the outcome of interest.

The challenge then is to incorporate the outcome information in a meaningful way that can help identify discriminative groups for a particular outcome. Previous studies have explored using residuals as the dependent variable and empirically assessed viability on classification and regression^36,37^. For largely censored samples, the use of residuals has the advantage that each subject would be associated with a residual regardless of its event status. This allows us to incorporate all data available into the training process.

Martingale residuals^36^ in particular can be interpreted as a measure of excess of deaths. Martingale residuals are defined as follows:

$${M}_{i}(t)={N}_{i}(t)-{\int }_{0}^{t}{Y}_{i}(s){e}^{\beta {\prime} {Z}_{i}(s)}d{\Lambda }_{o}(s)$$

*N*_*i*_(*t*) indicates the number of observed events at time t for subject. *Y*_*i*_(*t*) is a 0–1 process indicating whether the *i*^*th*^ subject is at risk at time *t*, *β* is a vector of regression coefficients, *Z*_*i*_(*t*) is a *p* dimensional vector of feature processes, and Λ_*o*_ is the baseline cumulative hazard function. Residuals are bounded between ∞ and +1.

**Figure 1:**
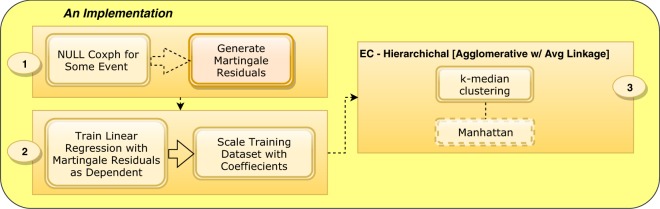
Supervised Scaling Approach. A null Cox model is trained in order to obtain a proxy dependent variable (1), e.g. martingale residuals. The fitted coefficients obtained from training a supervised learning method, e.g. linear regression, are used to scale our feature space (2). A clustering method is applied over the scaled feature space (3). The clustering implementation here shown is consensus clustering over 1k runs of the k-median (k = 2) clustering method using different initial seeds and Manhattan distance as the dissimilarity measure.

The Supervised Scaling processing pipeline is illustrated in Fig. 1. First, a null Cox model (i.e., one in which no covariates are included) is trained for a particular outcome in order to obtain a proxy dependent variable, the martingale residuals (1). Then, these residuals are used to train a linear regression model such that the fitted coefficients are used to scale the feature space (2). This effectively produce features weights associated to the outcome.

Finally, the scaled feature space is clustered using a machine learning algorithm, e.g. consensus clustering over 1000 runs of k-medians (3). Through the remainder of this paper, scaling or scaled refers to applying these feature weights in addition to first standardizing the features with min max normalization. Once we have clustered the data with Supervised Scaling, we proceed to use these cluster labels as a feature in the prediction method.

### Survival models

Since Cox proportional hazards (Cox) models are generally used to model survival and meaningful comparisons among models with various metrics can be made, we construct several Cox models using different features, including the cluster label where indicated, as described below.

- *AJCC Only* - Only 4 AJCC categories are considered in the model.
- *[Sc*.*] Cluster Only* - only the cluster label as a feature after standardizing and scaling of the feature space.
- *[Stand*.*] Cluster Only* - only the cluster label as a feature without scaling the feature space (only standardization).
- *Only AJCC* & *[Sc*.*] Cluster* - Only 4 AJCC categories and scaled feature space cluster labels are considered in the model.
- *Clin*. *Only* - only the clinical features.
- *Clin* & *X* - Clinical features and, in addition, what X describes (eg. *Rad*. for the 4 radiomic feature signature, *[SC*.*] Cluster Only* for the scaled feature space cluster labels, etc).

In addition to these Cox models, we also evaluated Random Survival Forest (RSF) implemented in the randomForest- SRC(v2.7) package^38^. The number of trees was set to 500. Both the clinical features and the radiomic signature were available for the RSF model. The number of variables randomly selected as candidates to split a node (mtry) and the number of data points in a terminal node (nodesize) were optimized using a grid search and out-of-bag (OOB) error. Variable mtry was varied from 1 to 10, and node size was varied from 1–10 and 10 to 100 in increments of 5. After training, the optimal values for mtry and nodesize were 5 and 5 for OS, and 2 and 5 for RFS.

### Evaluation metrics

#### Log rank test

The log rank test or chi-square statistic allows us to compare the survival distribution among groups. The p-value associated compares against the null hypothesis that no group has a different survival distribution from the rest (the null distribution of the test statistic is a chi-square distribution with *n* − 1 degrees of freedom).

We consider the following performance measures for evaluating the survival prediction models^39^ and for model comparison:

#### AIC and AICc

AIC is a unitless quantity can be used to compare fits between different parametric models using the same data^40,41^. It estimates the Kullback Leibler divergence which means lower values are better for AIC. $AIC=2p-2ln(\hat{L})$.

AICc was used to overcome overfitting due to small sample size and its formula is given by: $AICc=AIC+\frac{2{p}^{2}+2p}{n-p-1}$.

$\hat{L}$ is the model evaluated at the most likely set of parameters, *n* is the number of samples, and *p* is the number of estimate.parameters. An -∆AIC value of at least 3 is considered to be a meaningful difference.

#### Log-likelihood ratio test (LRT)

The ratio between the log-likelihood of the simpler model to the model with more parameters^42^. The anova.coxph^43^ function was used for the test.

$$LRT=-2lo{g}_{e}(\frac{{L}_{null}(\hat{{\rm{\theta }}})}{{L}_{alternative}(\hat{{\rm{\theta }}})})$$

The test statistic under the null hypothesis approximates a chi-squared random variable with degrees of freedom equal to the difference in the number of parameters of the null vs alternative model.

#### C-Index

The C-Index (i.e. probability of concordance) is a unitless quantitative measure of the discriminative strength of a model. The C-Index is identical to the area under ROC for binary outcomes^44^. It is the proportion of evaluable predicted pairs with the right survival order over all evaluable pairs. The evaluability of the pairs is determined from the censored status of the individuals. A pair in which both subjects are censored is not evaluable, A pair in which one is censored and the other uncensored is evaluable if censored survival time is greater than the uncensored survival time^45^. A pair in which both subjects are uncensored is evaluable.

#### Calibration

Nam-D’Agostino calibration test statistic is considered an important validation^46^ and was computed using deciles of predicted risk The purpose is to assess agreement between the number of individuals that are predicted with a certain probability and the actual proportion of individuals^47^. Under the null hypothesis of a well-calibrated model, the test statistic approximately follows a chi-square distribution with 8 degrees of freedom.

#### Brier

This measure serves as an indication of overall performance. It is a quadratic scoring rule that ranges from a very informative model at 0 to 0.25 for a non-informative model when the probability for the event is 50%^39^. For evaluating the survival probabilities at 5-years we use inverse probability of censoring weighting to account for the censored samples^47^.

#### AUC

The Receiver Operating Characteristic (ROC) curve plots sensitivity against specificity for consecutive cutoffs of the survival probability. AUC is the area below this curve.

#### Adjusted rand index

This index measures the agreement for every pair between the labels assigned by the AJCC stage and the labels of the cluster. The adjusted refers to a correction for chance assignment^48^.

## Results

Two clusters were identified and evaluated using 10-fold cross validation for OS and RFS.

### Radiomic feature selection

The top 4 radiomic features selected from RReliefF for both OS and also for RFS were:

- F25.ShapeVolume
- F29.IntensityDirectLocalRangeMax
- F5.IntensityDirectGlobalMax
- F29.IntensityDirectGlobalMax

### Clustering with supervised scaling

**Figure 2:**
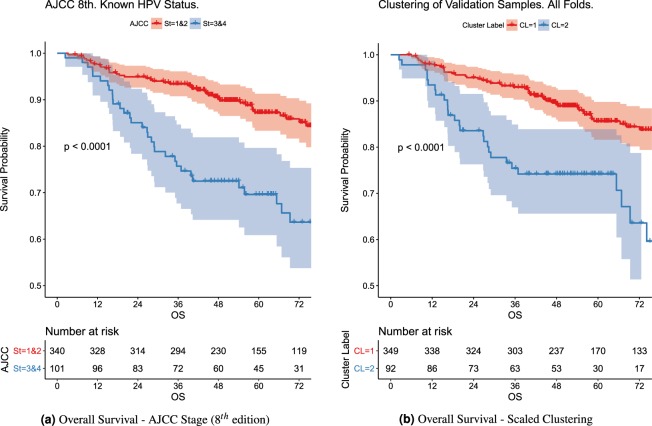
KM Curves for patients with known HPV Status. AJCC (8^*th*^ edition) KM curves are formed by aggregating AJCC stage categories as indicated by legend (Stage I and II vs Stage III and IV). The clustering of validation samples across folds likewise is only for known HPV Status in this comparison.

Figure 2 shows the KM curves for the cluster assignments over the validation samples across folds for the OS outcome.

The KM curves for the two clusters differ significantly (p-val < 0.0001). They are also significantly different (p-val < 0.01) for RFS. The demographic breakdown per cluster is given in Table 3 for OS and Table 4 for RFS. Albeit omitted for conciseness of figures and tables, for standardization only but not scaling, the p-values associated to the KM curve comparison were not significant for either outcome.

**Table 3:** Demographic breakdown per cluster for OS. Following AJCC standard definitions, T1 - T4: “Size and/or extent of the primary tumor”, Tx: “Primary tumor cannot be evaluated”, Tis: “Early cancer that has not spread to neighboring tissue”, and N0-N4: “Involvement of regional lymph nodes”.

OS Cluster Label Breakdown	*Cluster 1*			*Cluster 2*		
	Median or Frequency	(25th, 75th centiles) or Percent	Missing Frequency (Percent)	Median or Frequency	(25th, 75th centiles) or Percent	Missing Frequency (Percent)
Female						0
No	430	88.1		133	87.5	
Yes	58	11.9		19	12.5	
Age	0.5	(0.5, 0.7)		0.6	(0.5, 0.7)	0
HPV Status			0			0
Negative	29	5.9		21	13.8	
Positive	300	61.5		91	59.9	
Unknown	159	32.6		40	26.3	
T Category			0			0
T1,T2,Tis,Tx	340	69.7		66	43.4	
T3,T4	148	30.3		86	56.6	
N Category			0			0
N0, N1	270	55.3		69	45.4	
N2, N3	218	44.7		83	54.6	
Smoking Status			0			0
Current	105	21.5		32	21.1	
Former	178	36.5		60	39.5	
Never	205	42		60	39.5	
Smoking Pack Per Year (Current)	0.3	(0.2, 0.5)	10 (2)	0.3	(0.2, 0.5)	1 (0.7)
Tumor Subsite			0			0
BOT	245	50.2		82	53.9	
Tonsil	42	8.6		15	9.9	
GPS, NOS, Soft Palate	201	41.2		55	36.2	
White/Caucasian			0			0
No	35	7.2		21	13.8	
Yes	453	92.8		131	86.2	
Therapeutic			0			0
CC	267	54.7		72	47.4	
IC_and_CC	103	21.1		56	36.8	
IC_and_Radiation	52	10.7		8	5.3	
Radiation	66	13.5		16	10.5	
F25.ShapeVolume	0	(0, 0.1)	64 (13.1)	0.1	(0, 0.2)	20 (13.2)
F29.IntensityDirectLocalRangeMax	0.2	(0.2, 0.2)	64 (13.1)	0.2	(0.2, 0.3)	20 (13.2)
F5.IntensityDirectGlobalMax	0	(0, 0.1)	64 (13.1)	0.2	(0.1, 0.2)	20 (13.2)
F29.IntensityDirectGlobalMax	0	(0, 0.1)	64 (13.1)	0.2	(0.1, 0.3)	20 (13.2)
AJCC 8th						0
I	197	40.4		41	27	
II	82	16.8		27	17.8	
III	45	9.2		29	19.1	
IV	164	33.6		55	36.2	
OS Survival Time	72.8	(47.8, 100.9)	0	53.7	(35.1, 78.4)	0
OS Event Time (Uncensored)	41	(18.4, 69.2)	0	28.1	(15.3, 51.3)	0
Censored/Uncensored	400/88	82/18	0	108/44	71.1/28.9	0

BOT: Base of Tongue. NOS: Not otherwise specified. GPS: Glossopharyngeal Sulcus. CC: Concurrent Chemotherapy. IC: Induction Chemotherapy.

**Table 4:** Demographic breakdown per cluster for RFS.

RFS Cluster Label Breakdown	*Cluster 1*			*Cluster 2*		
	Median or Frequency	(25th, 75th centiles) or Percent	Missing Frequency (Percent)	Median or Frequency	(25th, 75th centiles) or Percent	Missing Frequency (Percent)
Female			0			0
No	366	88.6		193	86.5	
Yes	47	11.4		30	13.5	
Age	0.6	(0.5, 0.7)	0	0.5	(0.5, 0.7)	0
HPV Status			0			0
Negative	23	5.6		27	12.1	
Positive	262	63.4		127	57	
Unknown	128	31		69	30.9	
T Category			0			0
T1,T2,Tis,Tx	277	67.1		127	57	
T3,T4	136	32.9		96	43	
N Category			0			0
N0, N1	228	55.2		108	48.4	
N2, N3	185	44.8		115	51.6	
Smoking Status			0			0
Current	79	19.1		57	25.6	
Former	154	37.3		83	37.2	
Never	180	43.6		83	37.2	
Smoking Pack Per Year (Current)	0.3	(0.2, 0.5)	5 (1.2)	0.3	(0.2, 0.5)	6 (2.7)
Tumor Subsite			0			0
BOT	214	51.8		112	50.2	
Tonsil	30	7.3		25	11.2	
GPS, NOS, Soft Palate	169	40.9		86	38.6	
White/Caucasian			0			0
No	34	8.2		21	9.4	
Yes	379	91.8		202	90.6	
Therapeutic			0			0
CC	223	54		114	51.1	
IC_and_CC	95	23		63	28.3	
IC_and_Radiation	42	10.2		18	8.1	
Radiation	53	12.8		28	12.6	
F25.ShapeVolume	0	(0, 0.1)	57 (13.8)	0.1	(0, 0.1)	26 (11.7)
F29.IntensityDirectLocalRangeMax	0.2	(0.2, 0.2)	57 (13.8)	0.2	(0.2, 0.3)	26 (11.7)
F5.IntensityDirectGlobalMax	0	(0, 0.1)	57 (13.8)	0	(0, 0.2)	26 (11.7)
F29.IntensityDirectGlobalMax	0	(0, 0.1)	57 (13.8)	0.1	(0, 0.2)	26 (11.7)
AJCC 8th			0			0
I	164	39.7		73	32.7	
II	75	18.2		33	14.8	
III	41	9.9		33	14.8	
IV	133	32.2		84	37.7	
RFS Survival Time	62.7	(40.8, 96.8)	0	58.9	(32.6, 94.3)	0
RFS Event Time (Uncensored)	17.4	(10.8, 39.4)	0	17.6	(8.9, 33.4)	0
Censored/Uncensored	336/77	81.4/18.6	0	182/41	81.6/18.4	0

Following AJCC standard definitions, T1 - T4: “Size and/or extent of the primary tumor”, Tx: “Primary tumor cannot be evaluated”, Tis: “Early cancer that has not spread to neighboring tissue”, and N0-N4: “Involvement of regional lymph nodes”. BOT: Base of Tongue. NOS: Not otherwise specified. GPS: Glossopharyngeal Sulcus. CC: Concurrent Chemotherapy. IC: Induction Chemotherapy.

### Comparison with AJCC staging system (8th edition)

We compare the KM plots for to AJCC stage against the clustering label results mentioned previously as indicated in the same Fig. 2. To aid this comparison, Stages I and II were grouped together, likewise Stages III and IV were grouped together. The Adjusted Rand Index comparing the 2 clusters in these figures for OS vs the AJCC groupings is 0.193, and 0.104 for RFS. When comparing the cluster labels vs. all the 4 stages of AJCC considering the unknown HPV, it is 0.028 for OS and 0.023 for RFS. Given that this pairwise agreement measure is low, but we know that both (1) AJCC is clinically informative and moreover (2) that the clusters have a strong discrimination on the outcome, in the model comparison we compare how adding both the label and the AJCC status affects the model.

### Model comparisons and prediction

We compare how meaningfully the cluster labels are by quantitatively assessing them (AIC/AICc and LRT) as an additional feature in the Cox model as shown in Table 5. We consider the entire dataset and the cluster labels are those assigned to the validation samples at every fold.

**Table 5:** Model comparisons of various Cox models and AJCC varying the features.

*Vs*. *Clinical*	**OS**			**RFS**		
Model	**AIC**	**AICc**	**LRT**	**AIC**	**AICc**	**LRT**
Clin. & Rad.	+21.80	+21.35	5.36e-06	+17.82	+17.37	3.43e-05
Clin. & [Sc.] Cluster	+15.60	+15.50	2.72e-05	+7.03	+6.92	2.66e-03
Clin. & [Stand.] Cluster	+0.52	+0.42	1.12e-01	−1.88	−1.99	7.34e-01
Clin. & AJCC	−1.01	−1.34	1.73e-01	+2.05	+1.72	4.49e-02
Clin. & AJCC & [Sc.] Cluster	+13.47	+13.02	2.55e-04	+8.65	+8.19	2.26e-03
*Vs*. *NULL*	**OS**			**RFS**		
[Sc.] Cluster Only	+30.69	+30.68	1.08e-08	+12.89	+12.88	1.14e-04
[Stand.] Cluster Only	+1.77	+1.76	5.22e-02	−0.19	−0.20	1.79e-01
AJCC Only	+11.48	+11.44	5.64e-04	+8.93	+8.89	1.88e-03
Only AJCC & [Sc.] Cluster	+36.54	+36.48	4.96e-09	+19.50	+19.43	1.58e-05

The baseline model v*s*. *Clinical* refers to the Cox model using clinical covariates whereas for v*s*. *NULL* it refers to the *null* Cox model. Models were fitted on the entire dataset and the cluster labels (for the models the labels were used, i.e. denoted by **Cluster**) were those assigned to the validation samples at every fold. AIC/AICc values are given relative to the baseline model as the negated difference. **[Stand**.**]** Refers to min max standardization only. **[Sc**.**]** Refers to scaling features prior to clustering.

This table compares against two baseline models. To facilitate the comparison between the models, the results are displayed as the negated difference to these baseline models for AIC and AICc such that negative values indicate a worse model and positive values a better one relative to the baseline models. Table 5 compares against the baseline models, v*s*. *Clinical* considers a Cox model with only the clinical features and v*s*. *NULL* against the *null* Cox model (Cox model with no covariates). As the clinical features are known features that are relevant to prognosis, we also consider this model as a baseline. Moreover, since we know that AJCC is a clinically relevant categorization we consider it as a feature against both baseline models and compare it against our quantitative approach to categorization.

When considering the clusters formed only through standardization ([Stand.]) of the features, LRT and AIC indicate that these labels are not informative as features against either of the baseline models.

The models with overall better AICs (>3) vs. the clinical model were Clin & Rad. and the models using scaled clusters ([Sc.] Cluster) as features. This is expectedly more so against the *null* model. For the models with the [Sc.] Cluster as feature, the 95% CI for the estimated hazard ratio of the non-reference label was [2.22,4.66] for OS and [0.30,0.66] for RFS. Similarly, when considering the clinical features and the cluster label, the interval for the cluster label was [1.64,3.64] for OS and [0.34,0.78] for RFS. All hazard ratios for clusters with standardized only are non-significant. As expected from the fact that the AJCC labels do not match with the cluster labels yet both could be informative, when comparing against the *null* model we note that the inclusion of both AJCC and the [Sc.] Cluster reflects a better model with AIC rather than either [SC.] Cluster or AJCC alone. However, once we control for the clinical variables, AJCC does not indicate any significant improvement.

Additionally, even when controlling for AJCC and Clinical, the [Sc.] Cluster feature still provides significant hazard ratios for the non-reference label, which are [2.01,3.59] for OS and [0.35,0.80] for RFS.

Table 6 shows the main model prediction evaluation using four of the metrics described in the Evaluation Metrics section. With our proposed method, when evaluating the labels as a feature in Clin.&[Sc.] Cluster, for OS we see better values for AUC, Brier and C-Index, and a well calibrated model. As for RFS, using the 4 radiomic signature features shows the better AUC, Brier and C Index despite not being as well calibrated as the other models Clin.&[Sc.]. Clusters with standardization only, as expected from AIC and LRT evaluation, considerably underperform against the radiomics or scaled clusters.

**Table 6:** Validation metric summary with 10-fold cross validation for OS and RFS outcomes. Cox model was used for all methods except Random Surv Forest.

Method	AUC	Brier	C-Index	Calibration
**OS**				
Clin. Only	0.6029 ± 0.0299	0.1349	0.6616 ± 0.0254	12.11
Clin. & Rad.	0.6203 ± 0.0302	0.1325	0.6785 ± 0.0259	15.25
Clin. & [Sc.] Cluster	0.6335 ± 0.0298	0.1298	0.6851 ± 0.0252	13.80
Clin. & [Stand.] Cluster	0.6061 ± 0.0297	0.1344	0.6645 ± 0.0254	10.47
Random Surv Forest	0.6292 ± 0.0309	0.1307	0.6818 ± 0.0262	28.85
Clin. & AJCC	0.6056 ± 0.0299	0.1347	0.6643 ± 0.0256	17.00
Clin. & AJCC & [Sc.] Cluster	0.6359 ± 0.0298	0.1302	0.6881 ± 0.0254	26.15
**RFS**				
Clin. Only	0.6111 ± 0.0308	0.1378	0.6044 ± 0.0276	12.58
Clin. & Rad.	0.6639 ± 0.0302	0.1335	0.6408 ± 0.0278	25.60
Clin. & [Sc.] Cluster	0.6377 ± 0.0302	0.1354	0.617 ± 0.0274	18.39
Clin. & [Stand.] Cluster	0.6008 ± 0.0312	0.1387	0.5902 ± 0.0281	11.48
Random Surv Forest	0.6080 ± 0.0316	0.1352	0.6043 ± 0.0292	10.30
Clin. & AJCC	0.6185 ± 0.0312	0.1359	0.6103 ± 0.028	11.29
Clin. & AJCC & [Sc.] Cluster	0.6483 ± 0.0306	0.1340	0.6279 ± 0.0278	19.19

Description of methods given in the Survival Models section.

## Discussion

As our driving motivation is to find discriminative groups of oropharyngeal head and neck cancer patients, we evaluate the performance of the proposed approach (Supervised Scaled Clustering) in terms of the KM curves it generates, the model performance under AIC and LRT metrics, and the predictive performance in terms of AUC, C-index, Calibration, and Brier scores.

Figure 2 compares the KM curves for the cluster groups against the latest edition of the AJCC staging (8^*th*^ edition) for patients with known HPV status. As can be seen in Fig. 2, both AJCC staging and the proposed Supervised Scaling, significantly discriminates with respect to the patient’s time to event. Moreover, when evaluating the predictive performance of these classification schemes, the proposed Supervised Scaling clustering method outperforms AJCC staging. As can be seen in Table 5, the addition of AJCC staging has significant LRTs for all comparisons except for OS when compared to the model with clinical features. For AIC, however, including the AJCC staging only improves when compared (∆(AIC) > 3) against the null model. Compared to the Cox model with clinical features only, the scaled cluster labels have high significance in LRT for the OS outcome whereas AJCC is not significant. The AIC values for the additional scaled cluster labels over only clinical are much greater than 3, which indicates an improved model.

Additionally, given the low pairwise agreement between AJCC staging and the cluster labels (rand index <0.2), we notice that when we include both AJCC and the scaled cluster label, the resulting model outperforms the models built with either one alone. This suggests that the information captured by the two stratifications is complementary. Compared to the null model, the combination of AJCC stage and scaled clustering shows the best performance for both OS and RFS. Not surprisingly and due to their correlation, the inclusion of AJCC in the clinical model, which already includes T-category and N-stage, shows no improvement. There is improvement, however, when the scaled cluster is included in the clinical model. These lead us to conclude that the proposed approach does indeed find a clinically meaningful categorization, complementary to AJCC staging, that can be further explored in future analyses.

As can be seen in Table 6, the cluster labels resulting from the proposed approach (i.e. [Sc.] Cluster or scaled cluster labels) shows improved performance over AJCC staging across all metrics, except [Sc.] Cluster is only well calibrated (Calibration < 15.5) for OS, whereas Clin. & AJCC is only well calibrated for RFS.

The proposed approach summarizes a high dimensional space into a single covariate. Machine learning approaches for feature selection identify a small subset of highly predictive features given an outcome variable. For these experiments, we use RReliefF and selected four radiomic features. When comparing the model performance of the scaled cluster labels to the radiomic signature, we see better AIC and LRT values for the radiomic signatures, but better values for AUC, Brier and C-Index for the scaled clustering for the OS outcome. For OS, Clin & Rad and Clin & [Sc.] are both well calibrated. These are encouraging results given the fact we performed feature selection using the whole dataset (and the outcome information) as the training set. The proposed approach was able to generate a single covariate that represents the entire radiomic feature space and exhibits prognostic value for OS and RFS.

Cox proportional hazard models are widely interpretable and commonly used in the oncologic community for survival analysis. We evaluate the proposed approach when the cluster labels are incorporated into a Cox model. However, this approach is potentially extendable to parametric approaches with minor modifications and could represent an additional step, albeit one not heavily investigated in the current study. The utility of a future space reduction has the added value of avoiding significant overfitting, and this also has potential applications across a wider range of machine learning style approaches which incorporate right-censored variables.

A further advantage of using the scaled clustering approach is that missing data can be handled without imputation nor removal by computing the distance between the patient and cluster centroids using the known available features. However, a thorough evaluation of missing data’s effect and performance comparison with established methods for data imputation are needed.

Clustering approaches specific in the context of leveraging right-censored outcomes have been previously considered in the literature. In Bair & Tibshirani^49^, for a gene dataset, the outcome information is considered by computing the univariate Cox score for all potentially relevant features, and then selected the top k of them as input to a nearest shrunken centroid clustering method. This method uses the Cox score for feature selection but performs clustering using equal weights. In our case, supervised scaling provides a mean to weight the features according to a particular outcome. A weighted approach has been also proposed in Gaynor & Bair^50^. In this work, univariate Cox score is assessed for each feature, the score is then ordered, and ultimately the k largest features are selected. A weighted sparse clustering maximizes a weighted between-cluster sum of squares. This work uses the censored outcome directly which would be less effective for largely censored data as the one used in this study. In Chen *et al*.^51^, the area under the curve between survival curves is considered as a measure of dissimilarity. The samples are initially grouped by considering all possible combinations of the features being considered. KM curves are formed by the groupings, the area between the curves would be the measure of dissimilarity and hierarchical clustering is applied over these dissimilarity values. In this study the number of cases considered was approximately 110,000 and 4 factors. Given our vastly smaller sample size and the consideration of many more feature combinations, the KM curves would need to be initially constructed with very few samples, where most would be censored, such that the curves and by extension the area between the curves would not be meaningful.

For many parametric and semi parametric methods such as Cox, the number of features that can be considered, especially given the limitation on sample size, is constrained despite the availability of increasing number of potentially relevant features. A limitation for the generalization of this study is that even after vastly reducing the feature space of potential radiomic features to four or one (the cluster label), the number of features used within the Cox model exceeds the rule of thumbs of ten events per covariate in the model.

From a clinical perspective, a limitation of the current study is the dearth of real-time collected human papillomavirus data status on historical patients within the data set; we circumvented this by incorporating the previous corresponding staging categories where there was uncertainty about HPV status. However, it should be noted that this is a major etiologic feature of head and neck cancers, and necessarily meant that the robustness of our analyses which incorporated HPV data was reduced by this. We hope in future iterations to increase the size of our HPV data set and include external validation in these larger data sets which would be of significant value. We attempted to correct for this by using a rigorous cross validation approach which we hope should demonstrate the robustness of our findings across potentially generalizable clinical scenarios. However nonetheless, as with any radiomics approach, the extensibility or generalizability of our data to other head neck cancer databases is contingent upon their similarity to the patient characteristics, treatment profiles, and demographic information contained herein.

A natural extension of our approach would be to use clustering as a way to represent other high dimensional spaces related to the outcome such as genomics and other omics spaces, and then using these labels as potentially useful features in prognosis. Other directions for future work include further evaluation to identify the attribute-values that characterize the clusters, and the evaluation of different parameters or algorithms considered in the different stages of the proposed processing pipeline. For example, the type of model fitted that can scale the feature space, the type of clustering and dissimilarity measures considered, and moreover, other ways to incorporate or leverage these discriminating clusters beyond as an additional feature used in a Cox model.

## Data Availability

The datasets analyzed during the current study are available from Scientific Data^25^ and TCGA.
